# A comparative study of English and Japanese ChatGPT responses to anaesthesia-related medical questions

**DOI:** 10.1016/j.bjao.2024.100296

**Published:** 2024-06-14

**Authors:** Kazuo Ando, Masaki Sato, Shin Wakatsuki, Ryotaro Nagai, Kumiko Chino, Hinata Kai, Tomomi Sasaki, Rie Kato, Teresa Phuongtram Nguyen, Nan Guo, Pervez Sultan

**Affiliations:** 1 Department of Anesthesiology, Perioperative and Pain Medicine. Stanford University School of Medicine, Stanford, CA, USA; 2 Private Practice Group, Pacific Anesthesia Inc., Honolulu, HI, USA; 3 University of Pittsburgh Medical Center, Magee-Women's Hospital, PA, USA; 4 Department of Anesthesiology, Indiana University School of Medicine, Indianapolis, IN, USA; 5 Department of Anesthesiology, Showa University School of Medicine, Tokyo, Japan

**Keywords:** anaesthesia, artificial intelligence, ChatGPT, digital health

## Abstract

**Background:**

The expansion of artificial intelligence (AI) within large language models (LLMs) has the potential to streamline healthcare delivery. Despite the increased use of LLMs, disparities in their performance particularly in different languages, remain underexplored. This study examines the quality of ChatGPT responses in English and Japanese, specifically to questions related to anaesthesiology.

**Methods:**

Anaesthesiologists proficient in both languages were recruited as experts in this study. Ten frequently asked questions in anaesthesia were selected and translated for evaluation. Three non-sequential responses from ChatGPT were assessed for content quality (accuracy, comprehensiveness, and safety) and communication quality (understanding, empathy/tone, and ethics) by expert evaluators.

**Results:**

Eight anaesthesiologists evaluated English and Japanese LLM responses. The overall quality for all questions combined was higher in English compared with Japanese responses. Content and communication quality were significantly higher in English compared with Japanese LLMs responses (both *P*<0.001) in all three responses. Comprehensiveness, safety, and understanding were higher scores in English LLM responses. In all three responses, more than half of the evaluators marked overall English responses as better than Japanese responses.

**Conclusions:**

English LLM responses to anaesthesia-related frequently asked questions were superior in quality to Japanese responses when assessed by bilingual anaesthesia experts in this report. This study highlights the potential for language-related disparities in healthcare information and the need to improve the quality of AI responses in underrepresented languages. Future studies are needed to explore these disparities in other commonly spoken languages and to compare the performance of different LLMs.

The application of artificial intelligence (AI) to healthcare has resulted in development of remarkable advancements in recent years.^1^ One sector of AI, large language models (LLMs), has been a particularly unique area of interest because of its potential to convey medical information in a human-like capacity.^2^ The public has increasingly turned to LLM chatbots to answer medical questions.^3^

Anaesthesiology is one of the largest specialties within the field of medicine and provision of anaesthesia for surgery is common globally with an estimated 310 million operations occurring annually.^4^ Anaesthesiology is also a medical specialty where precision, rapid information retrieval, and multilingual support are essential. The utility of LLMs in anaesthesiology remains underexplored.^5^ Additionally, differences in quality of medical information among different languages within the same LLM are unknown.^6^

Open AI's ChatGPT is one of the most popular open-source LLM chatbots and currently has an estimated 180.5 million registered users.^7^ ChatGPT's ability to engage in natural language conversations and provide contextually relevant responses makes it an intriguing candidate for assisting patients in answering frequently asked questions (FAQs) in anaesthesiology. Additionally, ChatGPT has multilanguage capability. Although there have been several studies evaluating ChatGPT's performance in answering medical questions in English, there is a paucity of studies which have explored other languages.^8^, ^9^, ^10^, ^11^ However, no studies to date have evaluated ChatGPT's performance in answering commonly asked anaesthesiology questions in Japanese and how these answers compare with responses in English. English is the most spoken language and Japanese is the ninth most commonly spoken language in the world.^12^ Comparing the quality of responses to medical questions in two frequently used languages in the world would provide valuable insights into potential disparities that may exist within this popular chatbot. This would also help to identify potential sources for disparities in healthcare information which require rectification.

This study aims to compare the quality of responses of ChatGPT (version 3.5) in English *vs* Japanese when answering FAQs related to general anaesthesia. The primary outcomes were content quality (consisting of accuracy, comprehensiveness, and safety) and communication quality (consisting of understanding, empathy/tone, and ethics) in each language. Secondary outcomes were direct comparative analysis between the English and Japanese languages.

## Methods

This study received Institutional Review Board Exemption, Protocol 71447 from Stanford University School of Medicine. The study was conducted between 8 October 2023 and 4 September 2024. Ethical considerations and data privacy protocols were adhered to throughout the study.

### Participants

We recruited practicing clinical anaesthesiologists fluent in both English and Japanese, defined by professional working experience in English-speaking countries or self-reported language proficiency. Potential participants were approached and asked for their willingness to participate via the Japanese Society of Anaesthesiologists research working group via Slack (*n*=6) San Francisco, CA, USA) and the Japanese Anaesthesiologists LINE (Shinjuku, Tokyo, Japan) group (*n*=14). Slack and LINE are messaging apps which include Japanese anaesthesiologists who practice Anaesthesia in the USA (*n*=14). Potential participants were informed of the study's purpose, procedures, and confidentiality measures. Eight Japanese anaesthesiologists agreed to participate as evaluators in this study. Among these eight participants, five have clinical experience working in both Japan and the USA; the remaining three anaesthesiologists hold a Japanese medical license and are fluent in English and medical and practice in USA and Japan. All participants are native Japanese speakers.

### Study protocols

The English FAQs in this study were selected from the top 10 US anaesthesia residency/fellowship program websites as ranked by the US News Ranking.^13^ After identification of the FAQs, the authors selected 10 questions covering a breadth of content relevant to patient care. Two authors (KA and MS) then translated these English FAQs into Japanese. The translated version was listed in an integrated spreadsheet and circulated to all eight of the Japanese anaesthesiologists (SW, RN, HK, KC, TS, RK, KA, and MS) who finalised the Japanese FAQs after discussion using modified methodology recommended by McKenna^14^ and others.

Each question from our list of FAQs in English and Japanese was inputted to ChatGPT (version 3.5), using a new session on three separate occasions. We followed a non-sequential approach as the responses from ChatGPT are known to vary when the same question is asked multiple times. Each response was collected in to a secure Qualtrics survey (Qualtrics, River Park Drive, UT, USA) and shared electronically to the eight experts for evaluation (Supplementary material, Appendix 1).

### Measurements

Each response was evaluated on a 5-point Likert scale, ranging from ‘strongly disagree’ to ‘strongly agree’ across three content quality metrics: accuracy, comprehensiveness, safety, and three communication quality metrics: understanding, empathy/tone, and ethics. These metrics were agreed upon by a group consensus by the study authors after a series of discussions during the study design phase. Experts were also asked to compare the English version and the Japanese version, using a separate 5-point Likert scale, ranging from ‘English is much better’ to ‘Japanese is much better’. The primary aim of this study was to compare the overall quality (content and communication) of English and Japanese responses to FAQs related to anaesthesia. Secondary aims were to compare individual aspects of response content quality (accuracy, comprehensiveness, and safety), communication quality (understanding, empathy/tone, and ethics) between English and Japanese responses, and determine if experts preferred English or Japanese responses.

### Analysis

The Likert scale from ‘strongly disagree’ to ‘strongly agree’ was scored from 1 to 5 for analysis. Median and inter-quartile range were presented. For each item, the Friedman test was used to compare the score between English and Japanese groups with Bonferroni correction. Then accuracy, comprehensiveness, and safety items were combined to represent content quality and understanding, empathy/tone, and ethics items were combined to represent communication quality. The content quality and communication quality were compared between English and Japanese groups using the Friedman test with Bonferroni correction. For each question, the reviewer's comparison of English *vs* Japanese is presented with count and percentage. All statistical tests were done using STATA v. 14.0 (StataCorp, College Station, TX, USA).

## Results

The institutions of work among the evaluators are provided in the authors list. The mean age and mean (standard deviation) number of postgraduate years of anaesthesia experience were 39 (7.2) and 14.3 (7.3), respectively.

Complete responses were obtained by all evaluators. All three responses were collected within 2 weeks of survey delivery. No reminders were required for completion. Overall scores for content quality and communication quality were higher for English responses (Table 1). Significant differences were demonstrable for two of the overall scores of content quality (comprehensiveness and safety) in the first and second response. The overall understanding component score was also higher in the English compared with Japanese response. Median scores for individual components of content and communication quality are provided in the Supplementary material, Appendix 2. The ChatGPT responses to the same questions posed on three separate occasions did not differ significantly (Table 1).

**Table 1:** Performance summary of Chat GPT repsonses comparing English vs Japanese frequently asked questions to general anaesthesia.

	First response			Second response		
	English	Japanese	*P*-value	English	Japanese	*P*-value
**Content quality:**						
Accuracy	4 [4–5]	4 [3–4]	0.197	4 [4–5] (1–5)	4 [2–4] (1–5)	<0.001
Comprehensiveness	4 [3–4]	3 [2–4]	0.0001	4 [4–4] (1–5)	2 [2–4] (1–5)	<0.001
Safety	4 [3–5]	2 [1–3]	<0.001	3 [3–4] (1–5)	3 [3–4] (1–5)	0.005
Overall content quality:	4 [3–5]	3 [2–4]	<0.001	4 [3–4] (1–5)	3 [2–4] (1–5)	<0.001
**Communication:**						
Understanding	4 [4–5]	4 [2–4]	<0.001	4 [4–5] (2–5)	3 [2–4] (1–5)	<0.001
Empathy/tone	3 [3–4]	3 [3–4]	0.250	4 [3–4] (2–5)	3 [3–3] (1–5)	<0.001
Ethics	3 [3–4]	3 [3–4]	0.983	3 [3–4] (2–5)	3 [3–3] (1–5)	0.013
Overall communication:	4 [3–4]	3 [3–4]	0.001	4 [3–4] (2–5)	3 [3–4] (1–5)	<0.001
**Overall**						
(all questions combined)	4 [3–4]	3 [2–4]	<0.001	4 [3–4] (1–5)	3 [2–4] (1–5)	<0.001

	Third response		
	English	Japanese	*P*-value
**Content quality:**			
Accuracy	4 [4–4] (2–5)	4 [2–4] (1–5)	<0.001
Comprehensiveness	4 [3–4] (1–5)	4 [2–4] (1–5)	0.052
Safety	3 [3–4] (2–5)	3 [3–4] (1–5)	0.091
Overall content quality:	4 [3–4] (1–5)	3 [2–4] (1–5)	<0.001
**Communication:**			
Understanding	4 [4–4] (2–5)	4 [3–4] (1–5)	0.001
Empathy/tone	3 [3–4] (2–5)	3 [3–4] (2–5)	0.332
Ethics	3 [3–4] (2–5)	3 [3–4] (2–5)	0.506
Overall communication:	3 [3–4] (2–5)	3 [3–4] (1–5)	0.006
**Overall**			
(all questions combined)	4 [3–4] (1–5)	3 [3–4] (1–5)	<0.001

Median [inter-quartile range] (range); P<0.006 is considered significant with Bonferroni correction.

A summary of findings of expert comparisons between English *vs* Japanese responses is provided in Table 2. Among the eight participants, more than half indicate that overall, the English responses were superior to the Japanese responses in all three responses.

**Table 2:** English *vs* Japanese large language model comparison.

	First response	Second response	Third response
	*N*=8	*N*=8	*N*=8
**Q1, *n* (%)**			
English much better	6 (75)	6 (75)	0
English somewhat better	2 (25)	2 (25)	5 (62.5)
Same	0	0	3 (37.5)
Japanese somewhat better	0	0	0
Japanese much better	0	0	0
**Q2, *n* (%)**	1 (12.5) 1 (12.5) 5 (62.5) 0 1 (12.5)	2 (25) 6 (75) 0 0 0	
English much better			1 (12.5)
English somewhat better			2 (25)
Same			4 (50)
Japanese somewhat better			0
Japanese much better			0
Missing			1 (12.5)
**Q3, *n* (%)**	2 (25) 1 (12.5) 4 (50) 1 (12.5) 0	0 2 (25) 2 (25) 3 (37.5) 1 (12.5)	
English much better			0
English somewhat better			1 (12.5)
Same			3 (37.5)
Japanese somewhat better			3 (37.5)
Japanese much better			0
Missing			1 (12.5)
**Q4, *n* (%)**	3 (37.5) 2 (25) 0 2 (25) 1 (12.5)	4 (50) 2 (25) 2 (25) 0 0	
English much better			1 (12.5)
English somewhat better			2 (25)
Same			4 (50)
Japanese somewhat better			0
Japanese much better			0
Missing			1 (12.5)
**Q5, *n* (%)**	1 (12.5) 1 (12.5) 4 (50) 2 (25) 0	1 (12.5) 2 (25) 4 (50) 0 1 (12.5)	
English much better			1 (12.5)
English somewhat better			1 (12.5)
Same			5 (62.5)
Japanese somewhat better			0
Japanese much better			0
Missing			1 (12.5)
**Q6, *n* (%)**	0 3 (37.5) 4 (50) 1 (12.5) 0	1 (12.5) 3 (37.5) 4 (50) 0 0	
English much better			0
English somewhat better			1 (12.5)
Same			6 (75)
Japanese somewhat better			0
Japanese much better			0
Missing			1 (12.5)
**Q7, *n* (%)**		1 (12.5) 6 (75) 1 (12.5) 0 0	
English much better	0		1 (12.5)
English somewhat better	1 (12.5)		2 (25)
Same	5 (62.5)		3 (37.5)
Japanese somewhat better	1 (12.5)		1 (12.5)
Japanese much better	0		0
Missing	1 (12.5)		1 (12.5)
**Q8, *n* (%)**		0 1 (12.5) 6 (75) 1 (12.5) 0	
English much better	4 (50)		1 (12.5)
English somewhat better	0		4 (50)
Same	2 (25)		2 (25)
Japanese somewhat better	0		0
Japanese much better	1 (12.5)		0
Missing	1 (12.5)		1 (12.5)
**Q9**		1 (12.5) 3 (37.5) 4 (50) 0 0	
English much better	1 (12.5)		0
English somewhat better	3 (37.5)		1 (12.5)
Same	2 (25)		6 (75)
Japanese somewhat better	1 (12.5)		0
Japanese much better	0		0
Missing	1 (12.5)		1 (12.5)
**Q10**			
English much better	1 (12.5)	3 (37.5)	0
English somewhat better	2 (25)	4 (50)	4 (50)
Same	4 (50)	0	2 (25)
Japanese somewhat better	0	0	0
Japanese much better	0	0	0
Missing	1 (12.5)	1 (12.5)	2 (25)
**Overall**			
English much better	0	0	0
English somewhat better	5 (62.5)	5 (62.5)	6 (75)
Same	0	0	0
Japanese somewhat better	2 (25)	0	1 (12.5)
Japanese much better	0	0	0
Missing	1 (12.5)	3 (37.5)	1 (12.5)

The main findings from this study are that English responses to anaesthesia FAQs are superior to Japanese responses in terms of overall content and communication quality. Furthermore, experts preferred English to Japanese responses in the version of ChatGPT that was evaluated. These findings underscore the crucial role of language proficiency and the availability of training data in determining the performance of AI language models in specialised medical contexts. Furthermore, these findings highlight the potential for disparities in medical information for different languages, even within the same LLM.

## Discussion

### Clinical implications

Overall, the English version of ChatGPT provided adequate responses to FAQs relating to anaesthesiology. Both median scores of content quality and communication quality in the English responses were 4 (somewhat agree) in all three responses except for the third response's overall communication quality, compared with a median score of 3 (neither agree or disagree) in all three Japanese responses. This suggests that the English version of ChatGPT may be better suited for answering patient FAQs and the Japanese version appears to be of lower quality.^15^ Language barriers in healthcare are well described, and are known to be associated with reduced primary and prenatal care utilisation.^16^^,^^17^

The differences in quality of responses demonstrated in this study could impact patient experience and quality of medical care if LLMs such as ChatGPT become adopted widely by patients as health information sources. The availability of extensive training data in English, likely enables the English version of ChatGPT to have a deeper understanding of anaesthesiology topics and therefore facilitate provision of better-quality responses.

The language-dependent performance observed in this study highlights the importance of considering language origin when implementing AI language models in medical practice, particularly in specialised fields such as anaesthesiology. Although LLMs can be effective tools for medical professionals, their utility can vary significantly depending on the language of interaction. In cases where a language lacks extensive training data, as observed in Japanese in this study, AI models may exhibit limitations in comprehensiveness and understanding. This discrepancy poses challenges for adopting AI language models in linguistically diverse healthcare settings. To apply the current AI model to another language model, it may be desirable to use the English AI language model response and then translate it into another language to optimise the response quality.

### Addressing language discrepancies

Language diversity is a crucial consideration in the medical field, as patients can be from any race/ethnicity and should expect the same quality of information regardless of primary language.^1^ Our investigation into ChatGPT's performance in both English and Japanese suggests that it shows potential capacity to bridge language barriers and cater to the global anaesthesiology community if the quality of responses can be improved. Future LLMs could be developed to generate responses based on summaries of data from all available languages rather than a single language on the internet.

### Research implications

To address the language-related limitations identified in this study, several strategies can be considered, which require future study and evaluation. Increasing the volume and diversity of training data in underrepresented languages, such as Japanese, may help to enhance the quality of responses provided in that language. Collaboration between AI researchers, medical professionals, and linguists is essential to curate specialised datasets that capture the nuances of medical terminology in different languages.^2^ Second, leveraging transfer learning techniques could enable AI models to benefit from their proficiency in one language to improve performance in another. State of the art models on smaller datasets in specific languages could also help bridge language gaps effectively. These models should be trained on diverse datasets and designed to provide accurate and coherent responses across a range of languages.^18^

Finally, tailoring AI language models for specific medical domains, such as anaesthesiology, could help improve their performance in responding to anaesthesiology questions, regardless of language.

### Limitations

We used ChatGPT-3.5, the free version of ChatGPT, in this study so the generalisability of findings for newer versions of ChatGPT (example, version 4.0, a paid and advanced version of the language model), is unclear. However, we chose version 3.5 because of its availability and accessibility to the public, unlike version 4 which is available only through a paid subscription. Furthermore, we only evaluated one LLM chatbot, and evaluations of other LLM chatbots (such as Google's Bard or Microsoft Bing Chat) or medical specific chatbots (e.g. Med-PaLM 2) are warranted in this context.

This study primarily focused on the comparison of AI language models in Japanese and English, which are just two of the numerous languages spoken worldwide. The findings may not fully represent the performance of these models in languages with different linguistic structures or smaller training datasets. Furthermore, instead of asking the questions one by one, we posed all 10 questions to ChatGPT simultaneously. The performance of ChatGPT might differ if we were to ask shorter questions.^19^ However, our findings do highlight the potential differences in quality of responses that are likely to occur in other languages compared with English responses. In addition, the field of AI is rapidly evolving, and the performance of AI language models may have changed since the study's completion, with ongoing updates and improvements potentially affecting their suitability for medical applications.

Beyond the linguistic challenges and the AI language model evolution, this study highlights the broader applications of AI language models in anaesthesia and healthcare. The models' capacity to provide accurate and contextually relevant information presents opportunities to expedite answering patient FAQs that may otherwise necessitate a clinical consultation. However, experienced anaesthesiologists still need to verify the content generated by LLMs to ensure adequate standards of responses.

## Conclusions

In summary, the English version of ChatGPT outperformed the Japanese responses to anaesthesia-related FAQs in our study. Future studies are needed to explore how these potential disparities in medical information can be addressed and whether the different available LLMs provide a similar quality of responses.

## Authors’ contributions

Conceived of the presented idea: TN, PS.

Developed the survey: KA, MS, SW, KC, KH, TS, RK.

Verified the analytical/statistics methods: NG.

Discussed the results and contributed to the final manuscript: all authors.

## Declarations of interest

The authors declare that they have no conflicts of interest.

## Funding

National Heart, Lung, and Blood Institute (R01HL166253-01A1 to PS) and National Institute of Child Health and Human Development (U54HD113142-01 to PS).

## Footnotes

Appendix A Supplementary data to this article can be found online at https://doi.org/10.1016/j.bjao.2024.100296.
