# Supplementary material for: A comparative study of English and Japanese ChatGPT responses to anaesthesia-related medical questions
Source: BJA Open. 2024 Jun 14;10:100296. doi: 10.1016/j.bjao.2024.100296 (PMC11225650; doi:10.1016/j.bjao.2024.100296)
Supplement: Multimedia component 1 [file mmc1.docx]

**Appendix 1**

**ChatGPT study questions and answers**

**English questions**
Assume you are an anesthesiologist working in the operating room and assume anesthesia options are available to the patient. Please also assume the patient consents to any recommended therapy and assume the patient has no contraindications to any proposed therapies.

1. Could you please explain the different types of anesthesia to me?

2. I've heard of general anesthesia, but I'm not entirely sure what it involves. Could you explain how it works?

3. I've heard about “regional anesthesia” and “local anesthesia.” Could you explain what it is and how it works?

4. I've been told about monitored anesthesia care, or MAC. Could you help me understand what that is?

5. Who is going to be responsible for giving me my anesthesia during surgery? Will it be an anesthesiologist or a Certified Registered Nurse Anesthetist?

6. I have some pre-existing medical conditions and had some past complications with anesthesia. How will these be managed during my surgery?

7. What are some of the common side effects or risks I might have with anesthesia?

8. How long before my surgery should I stop eating or drinking?

9. How long will it typically take for me to recover from the effects of anesthesia after surgery?

10. How will my pain be managed both during and after the surgery or procedure?

**1^st^ ChatGPT English answer**

1.Types of Anesthesia:

There are several types of anesthesia, each tailored to the patient's needs and the nature of the surgery or procedure:

General Anesthesia: Involves inducing a controlled state of unconsciousness, during which you'll be completely unaware and won't feel any pain or discomfort.

Regional Anesthesia: Blocks pain sensation in a specific region of the body. Examples include epidural and spinal anesthesia.

Local Anesthesia: Numbs a small, specific area of the body. It's often used for minor procedures.

Monitored Anesthesia Care (MAC): Involves administering medications to make you comfortable and relaxed, while you remain conscious. It's often used for procedures where general anesthesia isn't necessary.

2.General Anesthesia:

General anesthesia involves a combination of medications to induce unconsciousness, amnesia, muscle relaxation, and pain control. It's administered through an intravenous line and inhaled gases. An anesthesiologist monitors your vital signs throughout the procedure to ensure your safety.

3.Regional Anesthesia and Local Anesthesia:

Regional Anesthesia: Involves injecting anesthetic medication near a cluster of nerves to numb a larger area of the body. It's commonly used for surgeries involving limbs or the lower abdomen.

Local Anesthesia: Used to numb a small, specific area of the body. It's typically administered via injection or topical application and is often used for minor procedures.

4.Monitored Anesthesia Care (MAC):

MAC involves administering medications to help you relax and stay comfortable while undergoing a procedure. You'll likely remain conscious, and the anesthesiologist or a nurse anesthetist will monitor your vital signs closely.

5.Anesthesia Providers:

Both anesthesiologists and Certified Registered Nurse Anesthetists (CRNAs) can administer anesthesia. The choice depends on the complexity of the procedure, the patient's medical history, and the policies of the healthcare facility.

6.Pre-existing Conditions and Past Complications:

Your medical history and past complications will be thoroughly reviewed by the anesthesia team. They will create a tailored plan to address any concerns and minimize risks during the surgery.

7.Common Side Effects and Risks:

Potential side effects and risks of anesthesia can include nausea, vomiting, sore throat, dizziness, and allergic reactions. There are also rare but serious risks such as breathing difficulties, heart problems, or adverse reactions to medications.

8.Fasting Before Surgery:

Typically, you will be instructed not to eat or drink anything for several hours (usually around 6 to 8 hours) before the surgery. This is to reduce the risk of aspiration, where stomach contents enter the lungs during anesthesia.

9.Recovery from Anesthesia:

The time it takes to recover from anesthesia varies depending on the type of anesthesia used and individual factors. Generally, you'll be closely monitored as you wake up from the anesthesia, and you'll be discharged from the recovery area once you are stable.

10.Pain Management:

Pain management during and after surgery can involve various techniques, including medication, nerve blocks, and epidurals. Your anesthesia team will work with your surgical team to ensure your pain is well managed.

**2^nd^ ChatGPT English answer**

1.Types of Anesthesia:

General Anesthesia: It involves rendering the patient unconscious and pain-free throughout the surgery. This is usually achieved through a combination of intravenous drugs and inhaled anesthetic gases.

Regional Anesthesia: This includes techniques like epidural and spinal anesthesia, where specific nerves or regions of the body are numbed to block pain signals. The patient remains conscious but does not feel pain in the targeted area.

Local Anesthesia: It involves numbing a small, specific area of the body, such as a tooth or a small surgical site, using an injection of local anesthetic.

2. General Anesthesia: During general anesthesia, the anesthesiologist administers medications that induce unconsciousness and prevent pain sensation. This allows the surgical team to perform procedures without the patient experiencing any discomfort or awareness.

3. Regional Anesthesia: Regional anesthesia techniques involve injecting local anesthetic near nerves or the spinal cord to block pain signals from reaching the brain. This can be used for surgeries involving the lower abdomen, pelvis, or extremities.

4. Monitored Anesthesia Care (MAC): MAC involves administering sedatives and pain medications to keep the patient comfortable and relaxed during a procedure. It is often used for minor surgeries or procedures where the patient does not require full anesthesia but still needs pain management and sedation.

5. Responsibility for Anesthesia: The responsibility for administering anesthesia can vary. In some cases, it may be an anesthesiologist who is a medical doctor specialized in anesthesia. In other cases, especially in certain settings like rural hospitals or outpatient centers, a Certified Registered Nurse Anesthetist (CRNA) may administer anesthesia under the supervision of an anesthesiologist or surgeon.

6. Managing Pre-existing Conditions and Complications: Your pre-existing medical conditions and past complications with anesthesia will be carefully reviewed and managed by the anesthesia team. They will tailor the anesthesia plan to minimize risks and ensure your safety during surgery.

7. Common Side Effects or Risks of Anesthesia: Some common side effects or risks of anesthesia include nausea and vomiting, sore throat (if a breathing tube is used), dizziness, confusion, and allergic reactions. Serious complications such as respiratory problems or anesthesia awareness (being aware during surgery) are rare but possible.

8. Pre-surgery Eating and Drinking: Typically, you will be instructed to stop eating solid food several hours before surgery to reduce the risk of aspiration (inhaling stomach contents into the lungs). The specific fasting guidelines will be provided by your healthcare team.

9. Recovery from Anesthesia: The time it takes to recover from anesthesia varies depending on the type of anesthesia used, the length of surgery, and individual factors. Generally, patients recover consciousness and basic functions within a few hours after surgery, but full recovery may take several hours to days.

10. Pain Management: Pain management during and after surgery is an essential part of your care. The anesthesia team will use a combination of medications such as opioids, non-opioid pain relievers, nerve blocks, and other techniques to control your pain and ensure your comfort during recovery.

**3^rd^ ChatGPT English answer**

1.Types of Anesthesia:

General Anesthesia: Puts you in a deep sleep, so you're unconscious and unaware of surgery. It's commonly used for major procedures.

Regional Anesthesia: Blocks pain in a specific region of your body, like an arm or leg. Examples include epidurals and nerve blocks.

Local Anesthesia: Numbs a small area for minor procedures, like stitches or dental work.

2. General Anesthesia:

It works by administering medications that make you unconscious and unable to feel pain. You're carefully monitored throughout to ensure safety.

3. Regional Anesthesia and Local Anesthesia:

Regional anesthesia blocks nerve signals in a specific area, often used for pain control during and after surgery. Local anesthesia numbs a small part of your body.

4. Monitored Anesthesia Care (MAC):

It's a type of anesthesia where you receive sedation and pain management while still being conscious. It's often used for minor surgeries or procedures.

5. Anesthesia Providers:

Anesthesiologists and Certified Registered Nurse Anesthetists (CRNAs) are qualified to administer anesthesia. The specific provider depends on the hospital's policies and the complexity of the procedure.

6. Managing Medical Conditions and Past Complications:

Your medical history and past anesthesia experiences will be carefully considered. Anesthesia plans can be adjusted to minimize risks and manage any complications.

7. Common Side Effects or Risks of Anesthesia:

Side effects may include nausea, sore throat, confusion, or muscle aches. Risks can include allergic reactions, breathing problems, or rare complications like nerve damage.

8. Preoperative Fasting:

Typically, you're asked to stop eating solid foods 8 hours before surgery and to stop drinking clear liquids 2 hours before. Follow your doctor's instructions closely.

9. Recovery from Anesthesia:

Recovery time varies but can range from a few hours to several days, depending on the type of anesthesia used, your health, and the surgery's complexity.

10. Pain Management:

Pain is managed with medications during and after surgery, tailored to your needs. Options include opioids, non-opioid pain relievers, nerve blocks, and epidurals. Physical therapy and other techniques may also be used for pain relief.

**Japanese questions**

あなたが手術室に勤務する麻酔科医で、患者に麻酔の選択肢があると仮定してください。また、患者は推奨される治療法に同意し、提案された治療法に禁忌はないと仮定してください。
1. 麻酔の種類について教えてください。
2. 全身麻酔という言葉は聞いたことがありますが、どのようなものなのかよくわかりません。どのようなものか説明してください。
3. 「区域麻酔」と「局所麻酔」について聞いたことがあります。どのようなもので、どのように作用するのか説明してもらえますか？
4. 監視下鎮静麻酔（MAC）について聞いたことがあります。どのようなものか説明してください。
5. 手術中の麻酔は誰が担当するのですか？麻酔科医ですか、それとも麻酔専門看護師ですか。
6. 持病があり、過去に麻酔で合併症を起こしたことがあります。手術中はどのように管理されますか。
7. 麻酔に伴う一般的な副作用やリスクにはどのようなものがありますか。
8. 手術の何日前から飲食を控えるべきですか？
9. 手術後、麻酔の影響から回復するまでに通常どのくらいかかりますか。
10. 手術中と手術後の痛みはどのように管理されますか？

**1^st^ ChatGPT Japanese answer**

１．麻酔の種類について教えてください。

麻酔は大きく分けて全身麻酔、局所麻酔、および区域麻酔の3つの種類に分類されます。それぞれの種類は、患者の状態や手術の種類によって選択されます。

2．全身麻酔という言葉は聞いたことがありますが、どのようなものなのかよくわかりません。どのようなものか説明してください。

全身麻酔は、患者が意識を失い、痛覚や意識を感じなくなる状態を作り出す麻酔法です。投与される薬物によって、患者は手術中に痛みを感じず、手術を安全かつ快適に受けることができます。

３．「区域麻酔」と「局所麻酔」について聞いたことがあります。どのようなもので、どのように作用するのか説明してもらえますか？

区域麻酔は、特定の体の一部（例：腕や足）を麻酔する方法で、患者はその部位に痛みを感じませんが、他の部分は通常通り感じることができます。局所麻酔は、狭い範囲（例：歯や皮膚）に対して麻酔を行う方法で、その部位だけが無感覚になります。

４．監視下鎮静麻酔（MAC）について聞いたことがあります。どのようなものか説明してください。

監視下鎮静麻酔（Monitored Anesthesia Care, MAC）は、患者に軽度の鎮静と麻酔を提供しながら、患者が自分の呼吸や循環器系の状態を維持できるようにする麻酔法です。通常、局所麻酔や部分的な麻酔を補完するために使用されます。

５．手術中の麻酔は誰が担当するのですか？麻酔科医ですか、それとも麻酔専門看護師ですか。

手術中の麻酔は主に麻酔科医が担当します。麻酔科医は麻酔の専門家であり、適切な麻酔の管理と患者の安全を確保する役割を果たします。麻酔専門看護師も麻酔の管理を支援する役割を果たすことがあります。

６．持病があり、過去に麻酔で合併症を起こしたことがあります。手術中はどのように管理されますか。

持病や過去の麻酔合併症の情報は重要です。麻酔科医は患者の状態を評価し、過去の合併症や持病を考慮して麻酔計画を立てます。必要に応じて特別な注意や薬物調整を行い、安全な麻酔を提供します。

７．麻酔に伴う一般的な副作用やリスクにはどのようなものがありますか。

麻酔には各種のリスクが伴います。一般的な副作用には嘔吐、めまい、喉の痛みなどがあります。重篤な合併症としてはアレルギー反応、呼吸抑制、循環器系の問題などがありますが、麻酔科医はこれらのリスクを最小限に抑えるために注意を払います。

８．手術の何日前から飲食を控えるべきですか？

通常、手術の前夜から飲食を控える必要があります。麻酔の安全性と手術の成功に影響を及ぼす可能性があるため、手術前の指示に従うことが重要です。

９．手術後、麻酔の影響から回復するまでに通常どのくらいかかりますか。

回復の時間は手術の種類や麻酔の種類によって異なりますが、麻酔から完全に回復するまでには数時間から数日かかることがあります。麻酔の薬物が体内から排除され、患者の状態が安定するまで待つ必要があります。

10．手術中と手術後の痛みはどのように管理されますか？

手術中の痛みは麻酔によって抑制されます。手術後の痛みは、鎮痛薬や痛みの管理プランによって管理されます。患者の痛みの程度に応じて、麻酔科医や看護師が適切な薬物を使用して痛みを和らげます。

**2^nd^ ChatGPT Japanese answer**

1.麻酔の種類には、全身麻酔、局所麻酔、区域麻酔、脊髄麻酔などがあります。

2. 全身麻酔は、患者が意識を失い、無痛状態になる麻酔のことです。通常は薬物を静脈内に投与して行います。

3. 区域麻酔は、特定の体の部位を無痛状態にする麻酔で、局所麻酔はその中の一種です。局所麻酔は局所に薬物を注射して局所の神経を麻痺させますが、区域麻酔はより広い範囲を無痛状態にします。

4. 監視下鎮静麻酔（MAC）は、意識を保ちつつ、鎮静効果を与える麻酔の一種です。主に手術中や検査中に使用されます。

5. 手術中の麻酔は、通常は麻酔科医が担当しますが、麻酔専門の看護師も麻酔の管理や監視を行うことがあります。

6. 持病や過去の麻酔合併症がある場合、手術中は慎重に管理されます。麻酔チームはその情報を把握し、適切な麻酔プランを立てます。

7. 麻酔に伴う一般的な副作用やリスクには、嘔吐、喉の痛み、めまい、血圧の変動、呼吸の問題などがあります。

8. 手術前は通常、飲食を手術の8時間前から控えるように指示されますが、具体的な指示は医師や麻酔科医の指示に従います。

9. 手術後の麻酔からの回復には個人差がありますが、一般的には数時間から数日かかる場合があります。

10. 手術中の痛みは麻酔で管理され、手術後の痛みは鎮痛薬や適切な管理プランで管理されます

**3^rd^ ChatGPT Japanese answer**

1.麻酔の種類には主に以下のようなものがあります。

全身麻酔: 患者が意識を失い、無痛無感覚の状態になる麻酔です。

区域麻酔: 特定の部位や領域に麻酔を行い、その部位だけを無痛無感覚にします。

局所麻酔: 手術部位や周囲の局所組織に直接麻酔を行い、局所的な無痛無感覚を実現します。

監視下鎮静麻酔 (MAC): 患者は意識を保ちつつ、鎮静状態となります。

2. 全身麻酔は、患者が手術中やその後も意識を失い、無痛無感覚の状態になる麻酔です。通常は薬剤を静脈注射したり、吸入麻酔を行うことで実施されます。

3. 区域麻酔は、特定の部位や領域に麻酔を行い、その部位だけを無痛無感覚にします。例えば、脊髄麻酔や硬膜外麻酔などがあります。局所麻酔は、手術部位や周囲の組織に直接麻酔を行い、局所的な無痛無感覚を実現します。これには局所麻酔剤が用いられます。

4. 監視下鎮静麻酔 (MAC) は、患者が意識を保ちつつ、鎮静状態になる麻酔です。通常は薬剤を静脈注射して行います。この麻酔は、手術部位の局所麻酔や疼痛管理と併用されることがあります。

5. 手術中の麻酔は、麻酔科医が担当します。麻酔専門看護師も手術中に麻酔の管理や監視を補助する役割を果たすことがあります。

6. 持病があり、過去に麻酔で合併症を起こした患者の場合、手術中は患者の状態に応じて厳密に管理されます。事前に患者の健康状態や麻酔過敏症などの情報を収集し、適切な麻酔プランを立てます。

7. 麻酔に伴う一般的な副作用やリスクには、嘔気や嘔吐、頭痛、神経障害、呼吸器合併症などがあります。また、稀にアレルギー反応や麻酔中の意識喪失の問題も発生する可能性があります。

8. 手術の前日から飲食を控えるガイドラインは、一般的に手術前6～8時間は絶食が推奨されます。ただし、患者の健康状態や手術の種類によって異なる場合もあります。

9. 手術後、麻酔の影響から完全に回復するまでには、患者の状態や手術の内容によって異なりますが、一般的に数時間から数日かかる場合があります。

10. 手術中と手術後の痛み管理には、鎮痛薬や局所麻酔の使用、物理療法、心理的支援などが行われます。痛みの程度や患者の状態に応じて適切な管理方法が選択されます。

**Appendix 2. Performance of each question**

|  | **1^st^ answer** | | | **2^nd^ answer** | | |
| --- | --- | --- | --- | --- | --- | --- |
|  | **English** | **Japanese** | **p-value** | **English** | **Japanese** | **p-value** |
| **Q1**  Accuracy  Comprehensiveness  Understanding  Empathy / tone  Ethics  Safety | 4 [4-4.5]  5 [2-5]  4.5 [4-5]  3 [3-4]  3 [3-4]  4 [2.5-4.5] | 4 [3-4]  2 [2-2.5]  2 [1.5-3.5]  3 [2.5-3]  3 [3-3]  2.5 [1-3] | 0.414  0.025  0.014  0.046  0.180  0.103 | 4 [4-4.5] (2-5)  4 [4-4] (2-5)  4.5 [3.5-5] (2-5)  4 [3-4] (2-5)  3 [3-4] (3-4)  4 [3-4.5] (2-5) | 3.5 [2.5-4]  2 [2-4] (1-4)  2 [1-2] (1-3)  3 [2.5-3] (2-4)  3 [3-3] (1-4)  3 [2-3] (2-3) | 0.103  0.103  0.008  0.103  0.083 0.025 |
| **Q2**  Accuracy  Comprehensiveness  Understanding  Empathy / tone  Ethics  Safety | 4 [2.5-4]  4 [2-4]  4 [2.5-5]  3 [3-3.5]  3 [2.5-3]  3.5 [2.5-4.5] | 4 [4-4.5]  3 [2-4]  4 [2-4]  4 [2.5-4]  3 [3-3.5]  1.5 [1-3] | 0.317  0.414  0.318  0.157  0.317  0.103 | 4 [3.5-4.5] (2-5)  4 [3-4] (2-4)  4 [4-4.5] (4-5)  4 [3.5-4] (3-5)  3 [3-3.5] (3-4)  3 [2.5-4] (1-4) | 2 [2-2.5] (1-4)  2 [1-2] (1-3)  3 [2-4] (2-4)  3 [3-3] (3-4)  3 [3-3.5] (2-4)  3 [1.5-3] (1-4) | 0.005  0.005  0.008  0.014  0.564  0.083 |
| **Q3**  Accuracy  Comprehensiveness  Understanding  Empathy / tone  Ethics  Safety | 3 [2-4.5]  4 [2-4]  3.5 [1.5-5]  3 [2.5-3.5]  3 [2.5-3.5]  2.5 [1-4] | 3.5 [2-4]  2 [2-2.5]  2.5 [2-4]  3 [3-3.5]  3 [3-3.5]  2 [1-3.5] | 0.564  0.180  1.000  0.083  0.157  0.655 | 4 [2-4] (1-5)  3 [2-4] (2-5)  3.5 [3-4] (2-5)  3 [3-4] (2-5)  3 [3-3] (2-4)  3 [2.5-3] (1-5) | 4 [4-4] (3-5)  3.5 [2-4] (1-5)  4 [2-4] (2-5)  3 [3-3] (3-5)  3 [3-3.5] (3-5)  3 [3-4.5] (2-5) | 0.317  0.706  0.706  1.000  0.157  0.317 |
| **Q4**  Accuracy  Comprehensiveness  Understanding  Empathy / tone  Ethics  Safety | 4 [2.5-5]  4 [2.5-4.5]  4 [4-5]  3.5 [3-4]  3 [3-3.5]  3.5 [2.5-4.5] | 3.5 [1.5-4]  2.5 [1.5-3]  3 [1.5-4]  3 [3-3.5]  3 [3-3.5]  3.5 [2.5-4] | 0.480  0.257  0.317  0.317  1.000  1.000 | 4.5 [3-5] (2-5)  4 [3.5-4.5] (2-5)  4 [4-5] (4-5)  4 [3-4] (3-5)  3.5 [3-4] (3-5)  3 [3-3.5] (2-4) | 2 [2-2] (1-3)  2 [2-2] (1-3)  3 [2-3] (1-4)  3 [2-3] (2-5)  3 [3-3] (3-4)  3 [2-3] (1-4) | 0.014  0.008  0.008  0.083  0.046  0.180 |
| **Q5**  Accuracy  Comprehensiveness  Understanding  Empathy / tone  Ethics  Safety | 4 [3-4]  3 [2-4]  4 [3.5-5]  3 [3-4]  3 [2-3]  3.5 [2-4.5] | 4 [3-4.5]  3.5 [2-4]  4 [3-4.5]  3 [3-3]  3 [3-4]  2 [2-2.5] | 0.414  0.655  1.000  0.564  0.046  0.103 | 4 [4-4.5] (1-5)  4 [4-4.5] (1-5)  4 [4-5] (4-5)  3 [3-4] (3-5)  3.5 [3-4] (3-5)  3 [3-3.5] (1-4) | 4 [4-4.5] (2-5)  4 [3-4.5] (2-5)  4 [2-5] (2-5)  3 [3-3.5] (2-5)  3 [2.5-3] (2-5)  3 [3-3.5] (3-5) | 1.000  0.564  0.083  0.317  0.083  1.000 |
| **Q6**  Accuracy  Comprehensiveness  Understanding  Empathy / tone  Ethics  Safety | 4.5 [4-5]  4 [4-4.5]  5 [4-5]  4 [4-5]  3.5 [3-4]  4 [3.5-5] | 4.5 [4-5]  4 [4-4.5]  4 [2.5-4]  4 [2.5-4.5]  3 [3-4]  2 [1-2.5] | 0.564  0.317  0.014  0.157  0.317  0.025 | 4.5 [4-5] (4-5)  4 [4-4.5] (4-5)  4 [4-5] (4-5)  4 [4-5] (3-5)  3.5 [3-4] (3-5)  4 [3.5-4] (3-5) | 4 [4-4] (3-5)  4 [4-4] (2-5)  4 [3-4] (2-5)  3.5 [3-4] (2-5)  3 [3-4] (3-5)  3.5 [3-4] (3-5) | 0.083  0.157  0.046  0.083  0.564  0.157 |
| **Q7**  Accuracy  Comprehensiveness  Understanding  Empathy / tone  Ethics  Safety | 4 [4-5]  4 [2-5]  5 [4-5]  4 [3-5]  3 [3-4]  4 [2-5] | 5 [4-5]  4 [2-5]  4 [4-5]  4 [3-4]  3 [3-4]  2 [1-3] | 0.564  0.655  0.564  0.317  0.317  0.317 | 4 [3.5-5] (2-5)  4 [3-4] (1-5)  4 [4-5] (3-5)  3 [3-4] (3-5)  3 [3-3.5] (3-5)  3.5 [3-4.5] (1-5) | 4 [3-4] (1-5)  3 [2-4] (1-4)  3 [2.5-3.5] (2-5)  3 [2.5-3.5] (1-5)  3 [3-3] (3-5)  3 [3-4] (1-5) | 0.046  0.046  0.014  0.317  0.317  0.157 |
| **Q8**  Accuracy  Comprehensiveness  Understanding  Empathy / tone  Ethics  Safety | 4 [2-5]  4 [2-4]  5 [4-5]  4 [3-5]  3 [3-4]  4 [2-5] | 3 [2-4]  2 [2-2]  4 [2-5]  3 [3-3]  3 [3-3]  2 [1-4] | 0.103  0.059  0.083  0.083  0.317  0.103 | 2.5 [2-3.5] (2-4)  2 [2-3.5] (2-4)  4 [4-4] (2-5)  3 [3-4] (3-5)  3 [3-3.5] (3-5)  3 [2-3.5] (1-4) | 3.5 [2-4] (1-4)  2 [2-2] (2-5)  4 [3.5-4.5] (3-5)  3 [3-4] (3-5)  3 [3-4] (3-5)  3 [2-4] (1-5) | 0.564  1.000  0.564  1.000  0.317  1.000 |
| **Q9**  Accuracy  Comprehensiveness  Understanding  Empathy / tone  Ethics  Safety | 4 [4-5]  4 [3-4]  4 [4-5]  4 [3-4]  3 [3-4]  4 [4-4] | 4 [2-4]  2 [1-4]  4 [2-4]  3 [3-4]  3 [3-3]  3 [1-4] | 0.046  0.317  0.014  0.317  0.157  0.103 | 4 [4-4.5] (4-5)  4 [4-4.5] (2-5)  4 [4-5] (4-5)  3.5 [3-4] (3-5)  3 [3-4] (3-5)  4 [2.5-4] (2-4) | 4 [3-4] (2-4)  2.5 [2-4] (1-5)  3.5 [3-4] (2-5)  3 [3-4] (2-5)  3 [3-4] (3-5)  3 [2-3.5] (2-4) | 0.046  0.025  0.025  0.317  1.000  0.083 |
| **Q10**  Accuracy  Comprehensiveness  Understanding  Empathy / tone  Ethics  Safety | 5 [4-5]  4 [4-5]  5 [4-5]  4 [3-4]  4 [3-4]  4 [3-5] | 4 [3-4]  4 [3-4]  4 [4-5]  3 [3-4]  3 [3-4]  2 [1-3] | 0.046  0.046  0.157  0.317  0.157  0.046 | 4.5 [4-5] (4-5)  4.5 [4-5] (4-5)  4 [4-5] (4-5)  4.5 [3.5-5] (3-5)  3.5 [3-5] (3-5)  4 [3.5-4.5] (3.5-4) | 3 [2.5-3.5] (2-4)  2 [2-2.5] (1-3)  3 [2-3] (2-4)  3 [2.5-3] (2-5)  3 [3-3] (2-5)  3 [3-3] (2-5) | 0.008  0.005  0.005  0.014  0.083  0.025 |

Median [IQR]

p<0.0008 is considered significant with Bonferroni correction.

|  | **3^rd^ answer** | | |
| --- | --- | --- | --- |
|  | **English** | **Japanese** | **p-value** |
| **Q1**  Accuracy  Comprehensiveness  Understanding  Empathy / tone  Ethics  Safety | 4 [4-4] (2-5)  4 [3.5-4] (2-4)  4 [4-5] (4-5)  3 [3-3] (2-3)  3 [3-3] (3-4)  3 [3-3.5] (2-4) | 2.5 [2-4] (2-4)  3.5 [2-4] (2-5)  3 [2-4] (2-5)  3 [3-3] (2-4)  3 [3-3] (3-4)  3 [3-3] (2-3) | 0.025  0.655  0.025  0.317  1.000  0.317 |
| **Q2**  Accuracy  Comprehensiveness  Understanding  Empathy / tone  Ethics  Safety | 4 [4-4] (4-5)  4 [3-4] (1-5)  4 [3-4] (2-5)  3 [3-4] (3-4)  3 [3-4] (3-4)  3 [3-4] (2-4) | 2 [2-4] (2-4)  4 [3-4] (1-4)  4 [3-4] (2-5)  3 [3-3] (3-4)  3 [3-3] (3-4)  3 [3-4] (2-4) | 0.046  0.157  1.000  0.157  0.317  1.000 |
| **Q3**  Accuracy  Comprehensiveness  Understanding  Empathy / tone  Ethics  Safety | 4 [3-4] (2-4)  4 [2-4] (2-5)  3 [2-4] (2-5)  3 [3-3] (3-3)  3 [3-3] (3-3)  3 [3-3] (2-3) | 4 [3-4] (2-4)  4 [4-4] (4-5)  4 [3-4] (3-5)  3 [3-3] (3-4)  3 [3-3] (3-4)  3 [3-4] (3-4) | 0.564  0.083  0.317  0.317  0.317  0.157 |
| **Q4**  Accuracy  Comprehensiveness  Understanding  Empathy / tone  Ethics  Safety | 4 [2-4] (2-5)  3 [2-4] (2-5)  3 [3-4] (2-5)  3 [3-4] (3-5)  3 [3-4] (3-5)  3 [3-4] (2-5) | 2 [2-3] (1-4)  2 [2-4] (2-5)  2 [1-3] (1-4)  3 [3-3] (3-4)  3 [3-3] (3-4)  3 [3-3] (2-3) | 0.083  0.564  0.046  0.317  0.317  0.157 |
| **Q5**  Accuracy  Comprehensiveness  Understanding  Empathy / tone  Ethics  Safety | 4 [4-4] (2-4)  4 [3-4] (2-5)  4 [4-4] (2-5)  3 [3-4] (2-4)  3 [3-4] (2-4)  3 [3-4] (2-4) | 4 [2-4] (2-4)  4 [2-4] (1-4)  2 [2-4] (2-5)  3 [3-4] (2-5)  3 [3-4] (2-4)  3 [3-4] (2-4) | 0.317  0.180  0.157  0.317  1.000  1.000 |
| **Q6**  Accuracy  Comprehensiveness  Understanding  Empathy / tone  Ethics  Safety | 4 [4-4] (3-5)  4 [3-4] (2-4)  4 [4-4] (4-5)  4 [3-4] (3-4)  3 [3-4] (3-4)  4 [3-4] (2-4) | 4 [4-4] (2-4)  4 [3-4] (1-5)  4 [4-4] (2-5)  3 [3-4] (2-4)  3 [3-4] (2-4)  3 [3-4] (2-4) | 0.157  1.000  0.317  0.157  0.317  0.317 |
| **Q7**  Accuracy  Comprehensiveness  Understanding  Empathy / tone  Ethics  Safety | 4 [3-4] (2-5)  3 [2-4] (1-4)  4 [4-4] (3-5)  3 [3-4] (3-4)  3 [3-4] (3-4)  3 [3-4] (2-4) | 2 [2-4] (1-5)  2 [2-4] (1-5)  3 [2-4] (2-4)  3 [3-4] (3-4)  3 [3-4] (3-4)  3 [3-4] (2-4) | 0.317  1.000  0.046  0.317  0.317  0.317 |
| **Q8**  Accuracy  Comprehensiveness  Understanding  Empathy / tone  Ethics  Safety | 4 [4-5] (4-5)  5 [4-5] (4-5)  5 [4-5] (4-5)  3 [3-4] (3-4)  3 [3-4] (3-4)  4 [3-4] (3-5) | 4 [2-4] (1-5)  2 [2-4] (1-5)  4 [4-4] (1-5)  3 [3-4] (3-4)  3 [3-4] (2-4)  3 [2-3] (1-4) | 0.046  0.046  0.083  0.317  0.317  0.046 |
| **Q9**  Accuracy  Comprehensiveness  Understanding  Empathy / tone  Ethics  Safety | 4 [4-4] (4-5)  4 [3-4] (2-4)  4 [4-4] (4-5)  3 [3-4] (3-4)  3 [3-3] (3-4)  4 [3-4] (2-4) | 4 [4-5] (3-5)  4 [2-4] (2-5)  4 [4-5] (4-5)  3 [3-4] (3-4)  3 [3-4] (3-4)  4 [3-4] (2-5) | 1.000  1.000  0.317  0.317  0.157  0.317 |
| **Q10**  Accuracy  Comprehensiveness  Understanding  Empathy / tone  Ethics  Safety | 4 [4-4] (2-5)  4 [4-4] (2-5)  4 [4-4] (2-5)  3 [3-4] (3-5)  3 [3-4] (2-5)  3 [3-4] (2-5) | 4 [4-4] (2-4)  4 [3-4] (2-5)  3 [3-4] (2-5)  3 [3-4] (2-5)  3 [3-4] (2-5)  3 [3-4] (2-5) | 0.317  0.317  0.083  0.317  1.000  1.000 |

Median [IQR]

p<0.0008 is considered significant with Bonferroni correction.
